# Sleep duration and dietary macronutrient consumption can modify the cardiovascular disease for Korean women but not for men

**DOI:** 10.1186/s12944-015-0170-7

**Published:** 2016-01-27

**Authors:** Miae Doo, Yangha Kim

**Affiliations:** Department of Nutritional Science and Food Management, Ewha Womans University, 52, Ewhayeodae-gil, Seodaemun-Gu, Seoul, 120-750 Republic of Korea

**Keywords:** Cardiovascular disease, Dietary consumption, Framingham risk score, Sleep duration

## Abstract

**Background:**

Although the association between cardiovascular disease (CVD) and sleep duration is generally recognized, the results are inconsistent, and investigations examining the effects of seep duration and diet on CVD are rare.

**Methods:**

The gender-difference in the effect of the sleep duration on Framingham risk score (FRS)-related factors, 10‐year predicted CVD risk, and dietary consumption was analyzed in 14,111 subjects (Men *n* = 5,727; Women *n* = 8,384) aged ≥20 from the Korean National Health and Nutrition Examination Survey.

**Results:**

The gender difference in the CVD risk factors according to sleep duration was observed. Only women with short sleep durations (<7 h/day) exhibited elevated FRS factors, such as systolic blood pressures (SBP) (*P* < 0.001), diastolic blood pressures (DBP) (*P* = 0.008), and the proportion of hypertension (HTN) treatments (*P* < 0.001), but not for men. Moreover, the 10-year predicted CVD risk, as evaluated with the FRS, was higher in women with short sleep durations (*P* < 0.001). Women with short sleep durations consumed significantly more dietary carbohydrates (CHO) than those with normal sleep durations (*P* < 0.001). Additionally, the ORs for intermediate and high 10-year predicted CVD risks and CVD–related factors, such as high age, elevated SBP, and HTN treatment, significantly increased with short sleep durations among women [OR (95 % CI) = 1.709 (1.359–2.149) for CVD risk, 1.976 (1.756–2.224) for high age, 1.535 (1.291–1.826) for elevated SBP, and 1.515 (1.320–1.739) for HTN treatment].

**Conclusions:**

Short sleep duration influenced dietary carbohydrate consumption and elevated FRS-related factors as well as 10-year predicted CVD risk. Our findings demonstrated that the CVD risk has been potentially modified by short sleep durations and greater CHO consumptions.

## Background

Cardiovascular diseases (CVDs) are a group of heart and blood vessel disorders and are among the leading causes of death [1]. Recently, the incidence and prevalence of CVD have dramatically increased in Korea as they have worldwide [1, 2]. The risk factors for CVD include metabolic risk factors, such as hypertension, diabetes, hypercholesterolemia, and obesity, behavioral risk factors, such as smoking, an unhealthy diet, physical inactivity, heavy alcohol consumption, and gender, age, genetic disposition, and other factors [1].

Sleep duration is associated with adverse health outcomes, such as obesity [3], type 2 diabetes [4], hypertension [4], total mortality [5], and poor self-rated health [5]. Furthermore, it has generally been recognized that normal sleep durations decrease the risk of CVD events [6–12]. Although previous studies have reported results regarding CVD and sleep duration, these results are not consistent, and the associations of the risk of CVD with variations in sleep durations differ according to gender. Short sleep duration could cause a raise in total quantity and quality of dietary consumption [13, 14], then those change could eventually influence degree of the CVD [14]. However, studies of the relationship between CVD and sleep duration in Asian population are very rare.

The Framingham risk score (FRS) was used because its ease of application and superior equations for assessing 10‐year predicted CVD risks [15]. Therefore, the aim of this study was to examine how FRS -related factors and 10‐year predicted CVD risks are affected by different sleep durations among Korean adults who participated in the Korean National Health and Nutrition Examination Survey (KNHNES).

## Methods

### Study population and study protocol

Our study was based on data from the 5th KNHANES (2010–2012), which is a national cross-sectional survey that has been conducted periodically since 1998 by the Korea Centers for Disease Control and Prevention (KCDC) [16, 17]. The KNHANES has been performed to investigate health and nutritional statuses of the non-institutionalized civilian Korean population through systematic sampling with a multi-stage clustered probability design. The KNHANES consisted of a health interview, a health examination, and a dietary survey. From the total of 25,534 participants of the KNHANES over the age of 20 years, those who reported implausible daily energy consumptions (≤500 kcal or ≥ 5,000 kcal), those with incomplete sleep duration data, and those with FRS-related factors such as gender, age, systolic blood pressure (SBP), total cholesterol (TC), high density lipoprotein-cholesterol (HDL-C), smoking status, and hypertension (HTN) treatment were excluded. Finally, the remaining 14,111 participants were included in the analysis. The KCDC Institutional Review Board approved the survey protocol, and all participants provided written informed consent. However, this study did not require any ethics approval, because the KNHANES data are publicly available.

### Sleep duration assessment

Sleep duration was assessed using the following question: “How long do you usually sleep per day?’. Sleep duration of 7 h per day was taken as the reference point in accordance with previous studies [18, 19]; short sleep durations were defined as < 7 h per day, and normal sleep durations were defined as ≥ 7 h a day.

### FRS-related factors and definitions of the 10-year predicted coronary vascular disease risk

The 10‐year predicted CVD risk was evaluated according to the FRS. The FRS was calculated according to the following seven factors: gender, age, SBP, TC, HDL-C, smoking status, and HTN treatment. Blood pressure (BP) was measured with a mercury manometer with the subjects in a sitting position, and the average of two blood pressure readings was used for the analyses. Venous blood samples were collected after overnight fasting and used to measure the TC and HDL-C levels with an automatic analyzer 7600 (Hitachi, Tokyo, Japan). Smoking status and the medical history regarding HTN treatment were self-reported based on the health interview. To adjust for potential confounding variables, body mass indices (BMIs) were calculated by dividing the participants’ weights (kg) by their heights squared (m^2^) as measured according to a standardized procedure, also physical activity was assessed using the following question: “Do you experienced hard breath after moderate or high intensive exercise?”

The FRS was calculated using the following cutoffs after classification by age and gender [15]: SBP: < 120, 120–129, 130–139, 140–159, and ≥ 160 mmHg (also dependent on HTN treatment); TC: < 160, 160–199, 200–239, 240–279, and ≥ 280 mg/dl; HDL-C: < 40, 40–49, 50–59 and ≥ 60 mg/d; and smoking status: non-smoker and smoker. The 10-year predicted CVD risk (%) was calculated according to the total points from the FRS, and we also defined an intermediate/high 10-year predicted CVD risk as ≥ 10 %.

Age (≥50 years), SBP (>140 mmHg), TC (>200 mg/dl), HDL-C (<40 mg/dl), smoking status (smoker), and HTN treatment (yes) were used as CVD-related factors,.

### Dietary macronutrient consumption

The dietary survey was conducted by dietitians using the face-to-face interview method. In the dietary survey, the consumption of dietary macronutrients was assessed using a food frequency questionnaire that was developed and validated for the KNHANES [20].

### Statistical analysis

The statistical analyses were performed using the SPSS (version 21.0; IBM Corporation, Armonk, NY, USA) software for Windows. Sample weights form the KNHANES were used in all analyses to obtain the resulting estimates, which were representative of the entire Korean population. To evaluate general characteristics by gender, we used the *Pearson's chi-square* test to examine the categorical variables such as smoking status, HTN treatment, and physical activity, and the independent *t*-test to examine the continuous variables such as age, BMI, SBP, DBP, TC, HDL-C, 10-year predicted CVD risk, sleep duration, and dietary macronutrient intake. To analyze the effects of sleep duration on the 10‐year predicted CVD risk factors and dietary macronutrients intake, we used generalized linear models or multivariable logistic regression models after adjusting for covariates. BMI and physical activity were adjusted as covariate because they were reported to be associated with sleep duration and CVD, respectively [20, 21]. Moreover, multivariable logistic regression models were used to estimate the odds ratios (ORs) and 95 % confidence intervals (CIs) for the intermediate/high 10-year predicted CVD risk and CVD-related factors in reference to sleep duration ≥ 7 h per day after adjustments for covariates.

## Results

### General characteristics

The general characteristics stratified by gender are shown in Table 1. The average age and sleep duration per day were 44.85 years and 6.87 h, respectively. However, no difference was observed in the distributions of sleep duration by gender. SBP, DBP, smoking status, HTN treatment, 10-year predicted CVD risk, and physical activity were significantly higher in the men, but HDL-C was significantly higher in the women. The consumptions of all dietary macronutrients were significantly different according to gender.

**Table 1:** General characteristics in Koreans

	Total (*n* = 14,111)	Men (*n* = 5,727)	Women (*n* = 8,384)	*P*-value^*^
Age (years)	44.85 ± 0.24	44.02 ± 0.30	45.69 ± 0.25	<0.001
BMI (kg/m^2^)	23.70 ± 0.05	24.10 ± 0.06	23.30 ± 0.06	<0.001
SBP (mmHg)	117.98 ± 0.22	120.22 ± 0.27	115.74 ± 0.27	<0.001
DBP (mmHg)	76.64 ± 0.14	79.54 ± 0.21	73.74 ± 0.15	<0.001
TC (mg/dl)	188.40 ± 0.43	187.10 ± 0.64	189.70 ± 0.50	0.241
HDL-C (mg/dl)	52.62 ± 0.15	49.89 ± 0.22	55.36 ± 0.17	<0.001
Smoking status (%)	18.7	39.0	4.8	<0.001
HTN treatment (%)	20.5	21.4	20.0	0.014
10-year CVD risk (%)^a^	4.05 ± 0.07	6.40 ± 0.12	1.70 ± 0.05	<0.001
Sleep duration (hrs/d)	6.87 ± 0.14	6.88 ± 0.21	6.85 ± 0.19	0.907
Energy intake (kcal)	2058.89 ± 10.45	2395.09 ± 16.14	1722.70 ± 10.28	<0.001
Dietary protein (E %)	14.43 ± 0.06	14.53 ± 0.08	14.33 ± 0.06	0.017
Dietary fat (E %)	18.27 ± 0.12	18.62 ± 0.16	17.92 ± 0.15	0.001
Dietary CHO (E %)	67.31 ± 0.15	66.86 ± 0.20	67.76 ± 0.19	0.001
Physical activity (%)	48.4	51.5	45.3	<0.001

*BMI* Body mass index, *SBP* Systolic blood pressure, *DBP* Diastolic blood pressure, *TC* Total cholesterol, *HDL-C* High density lipoprotein cholesterol, *HTN* Hypertension, *CVD* Cardiovascular disease risk, *hrs/d* hours/day, *E %* percentage of energy, *CHO* Carbohydrate

The data were represented the means ± SE or *N* (%) about representative of the Korean population

^*^ *P*-values were obtained using *t*-test or *x* ^2^-test

^a^10-year CVD risk was evaluated according to the FRS, which was calculated 7 factors such as gender, age, SBP, TC, HDL-C, smoking, HTN treatment

### 10-year predicted CVD risk factors and dietary macronutrient consumption according to sleep duration

The 10-year predicted CVD risk factors and dietary macronutrient consumptions according to sleep duration after applying the adjustments for BMI and physical activity are shown in Tables 2 and 3. For both the men and women, the mean ages were higher among the subjects with short sleep durations than those with normal sleep durations after applying the adjustments for BMI and physical activity (*P* = 0.011 for the men and *P* < 0.001 for the women, Table 2). Gender-differences in the CVD risk factors according to sleep duration were observed. The SBP, DBP, HTN treatment, and 10-year CVD risk were significantly different for the women but not for the men. The women with short sleep durations exhibited elevated CVD risk factors, including SBP (117.3 ± 0.4 mmHg vs. 114.0 ± 0.3 mmHg, *P* < 0.001), DBP (74.2 ± 0.2 mmHg vs. 73.5 ± 0.2 mmHg, *P* = 0.008), and the proportion receiving HTN treatment (19.3 % vs. 12.6 %, *P* < 0.001). Moreover, the 10-year predicted CVD risk, which increased with the FRS, was higher for the women with short sleep durations (2.1 ± 0.1 % vs. 1.4 ± 0.1 %, *P* < 0.001).

**Table 2:** Ten‐year predicted CVD risk factors by sleep duration in Koreans

	Men (*n* = 5,727)			Women (*n* = 8,384)		
	<7 hrs/d (*n* = 2,304)	≥7 hrs/d (*n* = 3,423)	*P*-value^*^	<7 hrs/d (*n* = 3,381)	≥7 hrs/d (*n* = 5,003)	*P*-value^*^
BMI (kg/m^2^)	24.23 ± 0.09	24.01 ± 0.08	-	23.72 ± 0.09	23.04 ± 0.08	-
Age (years)	44.98 ± 0.41	43.75 ± 0.36	0.011	48.98 ± 0.35	43.25 ± 0.29	<0.001
SBP (mmHg)	120.54 ± 0.39	120.79 ± 0.33	0.608	117.29 ± 0.37	113.98 ± 0.31	<0.001
DBP (mmHg)	79.68 ± 0.30	79.45 ± 0.25	0.543	74.16 ± 0.21	73.52 ± 0.18	0.008
TC (mg/dl)	188.02 ± 0.97	187.97 ± 0.78	0.968	189.53 ± 0.74	188.33 ± 0.65	0.220
HDL-C (mg/dl)	49.88 ± 0.31	49.30 ± 0.26	0.128	55.87 ± 0.24	55.64 ± 0.23	0.475
Smoking status (%)	37.6	40.0	0.523	4.8	4.8	0.987
HTN treatment (%)	23.4	20.0	0.120	24.6	16.9	<0.001
10-year CVD risk (%)^a^	6.42 ± 0.17	6.49 ± 0.15	0.732	2.08 ± 0.08	1.35 ± 0.05	<0.001

The data were represented the means ± s.e.m or *N* (%) about representative of the Korean population

*CVD* Cardiovascular disease, *hrs/d* hours/day, *BMI* Body mass index, *SBP* Systolic blood pressure, *DBP* Diastolic blood pressure, *TC* Total cholesterol, *HDL-C* High density lipoprotein cholesterol, *HTN* Hypertension

^*^ *P*-values were obtained using general linear model or multivariate logistic model after adjustment for BMI and physical activity

^a^10-year CVD risk was evaluated according to the FRS, which was calculated 7 factors such as gender, age, SBP, TC, HDL-C, smoking, HTN treatment

**Table 3:** Dietary macronutrients intake by sleep duration in Koreans

	Men (*n* = 5,727)			Women (*n* = 8,384)		
	<7 hrs/d (*n* = 2,304)	≥7 hrs/d (*n* = 3,423)	*P*-value^*^	<7 hrs/d (*n* = 3,381)	≥7 hrs/d (*n* = 5,003)	*P*-value^*^
Energy (kcal)	2415.80 ± 24.04	2381.43 ± 20.14	0.250	1696.49 ± 16.39	1739.33 ± 11.62	0.023
Protein (E %)	14.31 ± 0.10	14.68 ± 0.10	0.009	14.31 ± 0.10	14.33 ± 0.07	0.875
Fat (E %)	18.82 ± 0.26	18.42 ± 0.18	0.199	17.15 ± 0.21	18.46 ± 0.19	<0.001
CHO (E %)	66.87 ± 0.31	66.91 ± 0.24	0.919	68.54 ± 0.26	67.21 ± 0.23	<0.001

The data were represented the means ± s.e.m or *N* (%) about representative of the Korean population

*hrs/d* hours/day, *E %* percentage of energy, *CHO* Carbohydrate

^*^ *P*-values were obtained using general linear model or multivariate logistic model after adjustment for BMI and physical activity

The men with short sleep durations consumed less dietary protein compared to those with normal sleep durations (14.3 ± 0.1 % vs. 14.7 ± 0.1 %, *P* = 0.009 in Table 3). However, among the women, the consumption of dietary energy and fat were lower (1696.5 ± 16.4 kcal vs. 1739.3 ± 11.6 kcal, *P* = 0.023 and 17.2 ± 0.2 % vs. 18.5 ± 0.2 %, *P* < 0.001, respectively), but the consumption of dietary carbohydrate (CHO) was higher (68.5 ± 0.3 % vs. 67.2 ± 0.2 %, *P* < 0.001) in the subjects with short sleep durations.

### Adjusted odds ratios for the 10-year predicted CVD risk and related factors according to sleep duration

Table 4 shows the ORs for the 10-year predicted CVD risk and related factors according to sleep duration after adjustments for BMI and physical activity. Only among the women with short sleep duration, the adjusted ORs for intermediate/high 10-year predicted CVD, which was defined by a FRS ≥ 10 %, and related factors, including old age, elevated SBP, and HTN treatment, were significantly increased (*P* < 0.001 for all). However, among the men, no significant differences were observed in the intermediate/high 10-year predicted CVD risk or the related factors according to sleep duration. After adjusting for confounding variables, the women with short sleep durations exhibited a significantly increase the intermediate/high 10-year predicted CVD risk compared with those with normal sleep durations [OR (95 % CI) = 1.709 (1.359–2.149), *P* < 0.001]. The ORs for the 10-year predicted CVD risk-related factors of high age (>50 years), elevated SBP (>140 mmHg), and HTN treatment (yes) were increased among the women with short sleep durations compared with the women with normal sleep durations [ORs (95 % CIs) = 1.976 (1.756–2.224) for high age, 1.535 (1.291–1.826) for elevated SBP, and 1.515 (1.320–1.739) for HTN treatment].

**Table 4:** Adjusted odds ratio for intermediate/high 10-year predicted CVD risk and related factors by sleep duration in Koreans

	Men (*n* = 5,727)	Women (*n* = 8,384)
	OR (95 % CI)	OR (95 % CI)
High age (>50 years)	1.007 (0.882–1.150)	1.976 (1.756–2.224)^a^
Elevated SBP (>140 mmHg)	1.053 (0.863–1.284)	1.535 (1.291–1.826)^a^
High TC (>200 mg/dl)	0.988 (0.855–1.143)	1.092 (0.973–1.226)
Low HDL-C (<40 mg/dl)	1.061 (0.895–1.257)	1.071 (0.904–1.270)
HTN treatment (yes)	1.141 (0.966–1.349)	1.515 (1.320–1.739)^a^
Smoking status (smoker)	0.957 (0.838–1.094)	1.002 (0.783–1.283)
10-year predicted CVD risk (intermediate and high risk)	0.995 (0.871–1.136)	1.709 (1.359–2.149)^a^

*OR* Odds ratio, *CI* confidence interval, *CVD* Cardiovascular disease, *FRS* Framingham risk score, *SBP* Systolic blood pressure, *TC* Total cholesterol, *HDL-C* High density lipoprotein cholesterol, *HTN* Hypertension

ORs (95 % CI) were calculated in reference to sleep duration ≥ 7 h per day using multivariate logistic regression after adjustment for BMI and physical activity (^a^< 0.0001)

## Discussion

In a relatively large population of men and women, sleep duration was found to influence the 10-year predicted CVD risk and CVD-related factors after adjusting for BMI and physical activity; however, these findings were observed only in the women and not in the men. Moreover, these results were consistent with the effects of sleep duration on the ORs for the intermediate and high 10-year predicted CVD risk and CVD–related factors only among the women.

Consistent with previous studies [6–12], this study revealed that sleep duration was related to CVD. However, the associations between sleep duration and the CVD risk reported in previous studies are inconsistent. Some previous studies [9, 10] have reported that CVD is significantly elevated in longer sleepers, whereas other studies [11, 12] have found that only shorter sleep durations are associated with CVD or that both shorter and longer sleep durations are independently associated with CVD [6–8]. In contrast, some studies [23] have reported no association of sleep duration with CVD risk. Moreover, although this study did not directly examine the role of sleep duration on the incidence of CVD events, the women with short sleep durations exhibited elevations in the FRS–related factors and the 10-year predicted CVD risk. These results extend the observations of two studies [6, 23] that found that poor sleep duration is related to the 10-year predicted CVD risk as calculated with the FRS. As mentioned previously, the FRS is easier to apply and uses better equations to assess the 10‐year predicted CVD risk [14]. Additionally, this study revealed that the ORs for an intermediate/high 10-year predicted CVD risk and CVD–related factors, such as old age, elevated SBP, and HTN treatment, were significantly elevated with short sleep durations.

Specifically, the gender differences in the effect of sleep duration on CVD or the risk of CVD observed in this study agree with the findings of other studies [8, 24]. These discrepancies were likely partially explainable based on sleep quality, lifestyle, psychological and physical factors, etc. Generally, women tend to exhibit lower sleep quality, higher rates of insomnia, and more complaints about difficulty falling asleep than men [25] due to psychological factors such as anxiety and depression [26]. Moreover, the effect of sleep duration on CVD or the risk of CVD is weaker for men, among whom the percentage of smokers is higher, compared with women among whom the percentage of smokers is lower [27]. Because smoking strongly influences cardiovascular events, it is difficult to distinguish other effects; i.e., smoking exerts a ‘mask effect’. Additionally, because physical activity is associated with sleep duration [28, 29] and cardiovascular events [28, 30] and could also be co-influence both of these factors, the association between short sleep duration and CVD or the risk of CVD was stronger in the women who engaged in relatively less physical activity than in the men [27].

Previous studies [14, 31] have reported that short sleep duration might lead to increased energy consumption via the consumption of energy-rich foods, particularly foods high in CHO. This study found that the women with short sleep durations exhibited increased CHO consumption and decreased fat consumption; this pattern is similar to those of the previous reports, although this report observed decreases in total energy consumption. These results may be partly explained by the fact that women tend to exhibit more emotional eating and are more likely to have sleep disorders causes by psychological factors compared with men.

Although interactions between dietary consumption and sleep duration with respect to CVD components and the 10-year predicted CVD risk were definitely not observed in this study, there are currently no plausible explanations for the mechanisms of these results. Several previous studies have suggested that short sleep durations are related to CVD risk via the following mechanisms: (a) obesity and disorders in glucose metabolism that are caused by changes in dietary consumption via alterations in circulating levels of leptin and ghrelin [4]; (b) low-grade inflammatory states due to increased levels of circulating leukocytes and cytokines [32]; and (c) HTN and dyslipidemia per se [34]. However, among the women in our study, short sleep durations and greater CHO consumptions were strongly associated with elevations in CVD components and the predicted 10-year CVD risk. Furthermore, it is reasonable to conclude that the women who consumed high proportions of CHO consumed relatively low proportions of protein and fat because dietary macronutrients were represented as proportions of the total energy consumed. One meta-analysis [34] reported that low-CHO diets reduce body weight and improve lipid profiles and that long-term low-CHO diets can decrease the incidence of CVD events in a manner comparable to that of low-fat diets. In contrast, a recent review [35] found that low-CHO diets have little or no influence on body weight and CVD risk. In agreement with other studies, only the absolute amount of CHO consumed was considered in the definition of a low-CHO diet. To explore the effect of low-CHO diets on CVD risk, dietary quality and type should be considered because the quality and type of the macronutrients consumed are very important. Generally, because CHO-rich foods are consumed as the main staples of the diets of the Korean population, the consumptions of specific CHO-rich foods and the overall dietary CHO consumption were higher than those of Americans, particularly among the women (50.5 % of the total kcals consumed by American women are composed of CHO) [36]. Furthermore, the 10-year predicted CVD risk in this study was defined according to the intermediate risk level of a FRS ≥10 % because the proportion of participants with a high 10-year predicted CVD risk as defined by a FRS ≥ 20 % was very low. Therefore, the results of studies of Korean populations are difficult to generalize to the other populations. These results provide a foundation for investigations of the associations between sleep duration, dietary consumption, CVD risk and the differences in these associations according to gender. Moreover, future studies will need to consider the effects of the quality and type of consumed macronutrients on the interaction between sleep duration and CVD risk.

## Conclusions

This study using KNHANE which is relatively large in sample size and a national representative, demonstrated that short sleep duration influenced with more consumed dietary carbohydrate and elevated FRS-related factors as well as 10-year predicted CVD risk. This findings demonstrated that the CVD risk has been potentially modified by short sleep durations and greater CHO consumptions.

## Abbreviations

BMI: body mass index
BP: blood pressures
CHO: carbohydrate
CVD: cardiovascular disease
DBP: diastolic blood pressures
FRS: Framingham risk score
HDL-C: high-density lipoprotein cholesterol
HTN: hypertension
KNHNES: Korean National Health and Nutrition Examination Survey
SBP: systolic blood pressures
SE: standard error
TC: total cholesterol

## References

[1] World Health Organization (2011). Global atlas on cardiovascular disease prevention and control.

[2] Korean Society of Lipidology and Atherosclerosis (2009). The treatment guidelines of dyslipimia.

[3] Xiao Q, Arem H, Moore SC, Hollenbeck AR, Matthews CE (2013). A large prospective investigation of sleep duration, weight change, and obesity in the NIH-AARP Diet and Health Study cohort. Am J Epidemiol.

[4] Cappuccio FP, D’Elia L, Strazzullo P, Miller MA (2013). Quantity and quality of sleep and incidence of type 2 diabetes: a systematic review and meta-analysis. Diabetes Care.

[5] Knutson KL, Van Cauter E, Rathouz PJ, Yan LL, Hulley SB, Liu K (2009). Association between sleep and blood pressure in midlife: the CARDIA sleep study. Arch Intern Med.

[6] Ford ES (2014). Habitual sleep duration and predicted 10-year cardiovascular risk using the pooled cohort risk equations among US adults. J Am Heart Assoc.

[7] Buxton OM, Marcelli E (2010). Short and long sleep are positively associated with obesity, diabetes, hypertension, and cardiovascular disease among adults in the United States. Soc Sci Med.

[8] Kronholm E, Laatikainen T, Peltonen M, Sippola R, Partonen T (2011). Self-reported sleep duration, all-cause mortality, cardiovascular mortality and morbidity in Finland. Sleep Med.

[9] Xie D, Li W, Wang Y, Gu H, Teo K, Liu L (2014). Sleep duration, snoring habits and risk of acute myocardial infarction in China population: results of the INTERHEART study. BMC Public Health.

[10] Cai H, Shu XO, Xiang YB, Yang G, Li H, Ji BT (2014). Sleep duration and mortality: a prospective study of 113 138 middle-aged and elderly Chinese Men and women. Sleep.

[11] Cappuccio FP, Cooper D, D’Elia L, Strazzullo P, Miller MA (2011). Sleep duration predicts cardiovascular outcomes: a systematic review and meta-analysis of prospective studies. Eur Heart J.

[12] Garde AH, Hansen ÅM, Holtermann A, Gyntelberg F, Suadicani P (2013). Sleep duration and ischemic heart disease and all-cause mortality: prospective cohort study on effects of tranquilizers/hypnotics and perceived stress. Scand J Work Environ Health.

[13] Brondel L, Romer MA, Nougues PM, Touyarou P, Davenne D (2010). Acute partial sleep deprivation increases food intake in healthy men. Am J Clin Nutr.

[14] Markwald RR, Melanson EL, Smith MR, Higgins J, Perreault L, Eckel RH (2013). Impact of insufficient sleep on total daily energy expenditure, food intake, and weight gain. Proc Natl Acad Sci U S A.

[15] Expert Panel on Detection, Evaluation, and Treatment of High Blood Cholesterol in Adult (2001). Executive summary of the third report of the national cholesterol education program (NCEP) expert panel on detection, evaluation, and treatment of high blood cholesterol in adults (Adult Treatment Panel III). JAMA.

[16] The fifth Korean National Health and Nutrition Survey (KNHANES V). https://knhanes.cdc.go.kr/knhanes/eng/index.do. (2015) Accessed 13 May 2015.

[17] Kweon S, Kim Y, Jang MJ, Kim Y, Kim K, Choi S (2014). Data resource profile: the Korea National Health and Nutrition Examination Survey (KNHANES). Int J Epidemiol.

[18] Bixler E (2009). Sleep and society: an epidemiological perspective. Sleep Med.

[19] Gallicchio L, Kalesan B (2009). Sleep duration and mortality: a systematic review and meta-analysis. J Sleep Res.

[20] Kim DW, Song S, Lee JE, Oh K, Shim J, Kweon S (2014). Reproducibility and validity of an FFQ developed for the Korea National Health and Nutrition Examination Survey (KNHANES). Public Health Nutr.

[21] Xiao Q, Keadle SK, Hollenbeck AR, Matthews CE (2014). Sleep duration and total and cause-specific mortality in a large US cohort: interrelationships with physical activity, sedentary behavior, and body mass index. Am J Epidemiol.

[22] Holliday EG, Magee CA, Kritharides L, Banks E, Attia J (2013). Short sleep duration is associated with risk of future diabetes but not cardiovascular disease: a prospective study and meta-analysis. PLoS One.

[23] Matthews KA, Strollo PJ, Hall M, Mezick EJ, Kamarck TW, Owens JF (2011). Associations of Framingham risk score profile and coronary artery calcification with sleep characteristics in middle-aged men and women: Pittsburgh Sleep SCORE study. Sleep.

[24] Meisinger C, Heier M, Löwel H, Schneider A, Döring A (2007). Sleep duration and sleep complaints and risk of myocardial infarction in middle-aged men and women from the general population: the MONICA/KORA Augsburg cohort study. Sleep.

[25] van den Berg JF, Miedema HM, Tulen JH, Hofman A, Neven AK, Tiemeier H (2009). Sex differences in subjective and actigraphic sleep measures: a population-based study of elderly persons. Sleep.

[26] van Mill JG, Hoogendijk WJ, Vogelzangs N, van Dyck R, Penninx BW (2010). Insomnia and sleep duration in a large cohort of patients with major depressive disorder and anxiety disorders. J Clin Psychiatry.

[27] Magee CA, Kritharides L, Attia J, McElduff P, Banks E (2012). Short and long sleep duration are associated with prevalent cardiovascular disease in Australian adults. J Sleep Res.

[28] Hjorth MF, Chaput JP, Damsgaard CT, Dalskov SM, Andersen R, Astrup A (2014). Low physical activity level and short sleep duration are associated with an increased cardio-metabolic risk profile: a longitudinal study in 8–11 year old Danish children. PLoS One.

[29] Cho KO (2014). Sleep duration and self-rated health are independently associated with physical activity level in the Korean population. Iran J Public Health.

[30] Chomistek AK, Manson JE, Stefanick ML, Lu B, Sands-Lincoln M, Going SB (2013). Relationship of sedentary behavior and physical activity to incident cardiovascular disease: results from the Women’s Health Initiative. J Am Coll Cardiol.

[31] Haghighatdoost F, Karimi G, Esmaillzadeh A, Azadbakht L (2012). Sleep deprivation is associated with lower diet quality indices and higher rate of general and central obesity among young female students in Iran. Nutrition.

[32] Suarez EC (2008). Self-reported symptoms of sleep disturbance and inflammation, coagulation, insulin resistance and psychosocial distress: evidence for gender disparity. Brain Behav Immun.

[33] Steptoe A, Peacey V, Wardle J (2006). Sleep duration and health in young adults. Arch Intern Med.

[34] Hu T, Mills KT, Yao L, Demanelis K, Eloustaz M, Yancy WS (2012). Effects of low-carbohydrate diets versus low-fat diets on metabolic risk factors: a meta-analysis of randomized controlled clinical trials. Am J Epidemiol.

[35] Naude CE, Schoonees A, Senekal M, Young T, Garner P, Volmink J (2014). Low carbohydrate versus isoenergetic balanced diets for reducing weight and cardiovascular risk: a systematic review and meta-analysis. PLoS One.

[36] Wright JD, Wang CY (2010). Trends in intake of energy and macronutrients in adults from 1999--2000 through 2007--2008. NCHS Data Brief. 49.

